# Evolutionary genetics of flipper forelimb and hindlimb loss from limb development-related genes in cetaceans

**DOI:** 10.1186/s12864-022-09024-3

**Published:** 2022-12-02

**Authors:** Linxia Sun, Xinghua Rong, Xing Liu, Zhenpeng Yu, Qian Zhang, Wenhua Ren, Guang Yang, Shixia Xu

**Affiliations:** Jiangsu Key Laboratory for Biodiversity and Biotechnology, College of Life Sciences, Nanjing Normal University, Nanjing, 210023 China

**Keywords:** Cetaceans, Limb-related genes, Cis-regulatory elements, Flipper-forelimb, Hindlimb loss

## Abstract

**Background:**

Cetacean hindlimbs were lost and their forelimb changed into flippers characterized by webbed digits and hyperphalangy, thus allowing them to adapt to a completely aquatic environment. However, the underlying molecular mechanism behind cetacean limb development remains poorly understood.

**Results:**

In the present study, we explored the evolution of 16 limb-related genes and their cis-regulatory elements in cetaceans and compared them with that of other mammals. *TBX5*, a forelimb specific expression gene, was identified to have been under accelerated evolution in the ancestral branches of cetaceans. In addition, 32 cetacean-specific changes were examined in the SHH signaling network (*SHH*, *PTCH1*, *TBX5*, BMPs and *SMO*), within which mutations could yield webbed digits or an additional phalange. These findings thus suggest that the SHH signaling network regulates cetacean flipper formation. By contrast, the regulatory activity of the *SHH* gene enhancer—ZRS in cetaceans—was significantly lower than in mice, which is consistent with the cessation of *SHH* gene expression in the hindlimb bud during cetacean embryonic development. It was suggested that the decreased SHH activity regulated by enhancer ZRS might be one of the reasons for hindlimb degeneration in cetaceans. Interestingly, a parallel / convergent site (D42G) and a rapidly evolving CNE were identified in marine mammals in *FGF10* and *GREM1,* respectively, and shown to be essential to restrict limb bud size; this is molecular evidence explaining the convergence of flipper-forelimb and shortening or degeneration of hindlimbs in marine mammals.

**Conclusions:**

We did evolutionary analyses of 16 limb-related genes and their cis-regulatory elements in cetaceans and compared them with those of other mammals to provide novel insights into the molecular basis of flipper forelimb and hindlimb loss in cetaceans.

**Supplementary Information:**

The online version contains supplementary material available at 10.1186/s12864-022-09024-3.

## Background

Cetaceans (whales, dolphins, and porpoises) underwent a series of adaptive changes in morphology from their terrestrial ancestors as they returned to the aquatic environment from the land. Extant cetaceans that were supposed to originate from terrestrial artiodactyla were subdivided into two suborders: Mysticeti and Odontoceti [1]. One of the most consequential changes in cetaceans was the streamlined body shape, a result of the flipper forelimb and degenerated hindlimb, which generates lift and stabilizes the body as it maneuvers in the water [1, 2]. The flipper has a shortened arm, forearm bones, and webbed digits [2]. Other aquatic mammals also have flippers, such as pinnipeds (sea lions, seals and walruses) and sirenians (manatees and dugongs). Cetacean flippers operate as rudders while sirenians use their flippers to crawl along the riverbed. In contrast, pinnipeds have both fore and hind flippers for locomotion on land and in water, and adaptation to their amphibious lifestyle. For example, sea lions use their fore and hind flippers to walk on the land and support their body, whereas seals move on land via a dorsoventral body wave instead of limbs [3].

Cetacean forelimbs evolved a unique hyperphalangy: an increased number of phalanges in a single digit beyond the plesiomorphic condition of 2/3/3/3/3 for general mammals [4–7]. The number of phalanges per digit varies among cetaceans, from four or five phalanges to six or more in those with an extreme form of hyperphalangy [7]. For example, the long-finned pilot whale (*Globicephala melaena*) was reported to have 11–17 phalanges per digit [4]. Hyperphalangy creates a different shape and smooth edge flipper contour to distribute force across the flipper. Narrow and elongated flippers are used to swim fast, while broad-shaped flippers help to turn at slow speeds [7]. Recent identification shows that many genes are responsible for the formation of hyperphalangy, such as *WNT9A* (Wnt family member 9a) and several genes included in the *SHH* (Sonic hedgehog) pathway. For example, *WNT9A* is reportedly involved in interphalangeal joints, as a misexpression of *WNT9A* can prevent the joint from forming in chick limbs [8]. WNT9A protein is expressed in all developing interphalangeal joints in the pantropical striped dolphin (*Stenella attenuate*) [9]. SHH is produced in mesenchyme cells of the polarizing region at the posterior margin of the limb bud, and plays a key role in specifying digit identifies during vertebrate limb patterning and growth progress [10, 11]. Previous study revealed that SHH protein could induce digit duplication in the chick wing when SHH-expression cells were implanted beneath the anterior apical ectodermal ridge (AER) [12]. However, chick digits failed to form in wing and leg in the absence of SHH signaling, while *SHH*-deficient mouse limbs showed a great morphological change as only fused zeugopods were form and lacked the digit arch [11, 13, 14].

Lack of cell apoptosis is another characteristic of the development process for flippers [7]. As target protein of SHH signaling, BMP proteins (including BMP2, BMP4 and BMP7) can regulate interdigital cell apoptosis, resulting in digit separation [15]. Of note, *BMP7* is necessary for interdigital programmed cell death because interdigit-specific inactivation of *BMP7* led to syndactyly. In addition, *BMP2* and *BMP4* were reported to indirectly regulate interdigital cell death as indicated by the elimination of both *BMP2* and *BMP4* in the limb AER leading to reduce programmed cell death and thus webbed digits [16]. On the contrary, fibroblast growth factors (FGFs) members have been identified to as survival factors to reduce interdigital cell death in the limb mesoderm [17]. Correlative evidence suggests that *FGF8* is likely to be the most important factor performing this function [18]. Another member of FGFs family, *FGF10* is of vital importance in maintaining the expression of *FGF8* in AER. *FGF10* knockout mice die shortly after birth due to the complete absence of lungs, forelimbs and hindlimbs [19]. In addition, the T-box transcription factor genes *TBX4* and *TBX*5 are highly expressed during hindlimb and forelimb development, respectively [20, 21]. Paired-like homeodomain 1 (*PITX1*) is required for hindlimbs-specific patterning and development because the deletion of *PITX1* led to hindlimb malformation, such as tibial hemimelia and mirror-image polydactyly [22].

There is strong evidence that cis-regulatory elements, such as enhancers and silencers, are essential for orchestrating precise gene expression patterns [23]. In recent years, more and more conserved noncoding elements (CNEs) have been identified in genome-wide comparisons and confirmed to be regulatory elements. A classic example is that a 17-bp snake-specific deletion in ZPA (zone of polarizing activity) regulation sequence (ZRS)—an enhancer of *SHH*—resulted in ZRS deactivation, which contributed to limb loss during in the snake lineage [24]. In another example, the 17-bp snake-specific deletion in ZRS was reported to disrupt HOXD13 binding sites required for enhancer activity that diminished transcription of SHH in python hindlimb buds [25]. In addition, approximately 700 bp upstream of ZRS (“preZRS”) was found to have a point mutation that led to thumb-polysyndactyly syndrome in family members [26]. Similarly, HLEB (Hindlimb enhancer B), an enhancer of *TBX4*, was knocked out in mice, causing hindlimb defects [27]. In sticklebacks, *PITX1* enhancer—*pel*—led to pelvic loss because it prevented *PITX1* expression [28].

Although there are descriptions for the morphological adaptions of flipper forelimb and hindlimb loss in cetaceans, the underlying genetic basis is poorly understood. Here, we first examined the evolution pattern of 16 limb development-related genes in mammalian lineages: FGFs (*FGF8* and *FGF10*), BMPs (*BMP2*, *BMP4* and *BMP7*), members of the SHH signal pathway (*SHH*, *PTCH1*, S*MO*, *WNT9A* and *GREM1*) and transcription factors genes (including *TBX4*, *TBX5*, *PITX1*, *HAND2*, *LMBR1* and *HOXA13*). Second, we assessed whether accelerated evolution or sequence changes in regulatory elements targeting these limb development-related genes occurred in cetaceans. Third, we tested the effective activity of regulatory elements with unique changes via a cell-based functional assay. Finally, parallel / convergent sites were detected among marine mammals to explore whether genetic convergence occurred in flipper formation.

## Results

### Positive selection in limb-related genes in cetaceans

**Fig. 1:**
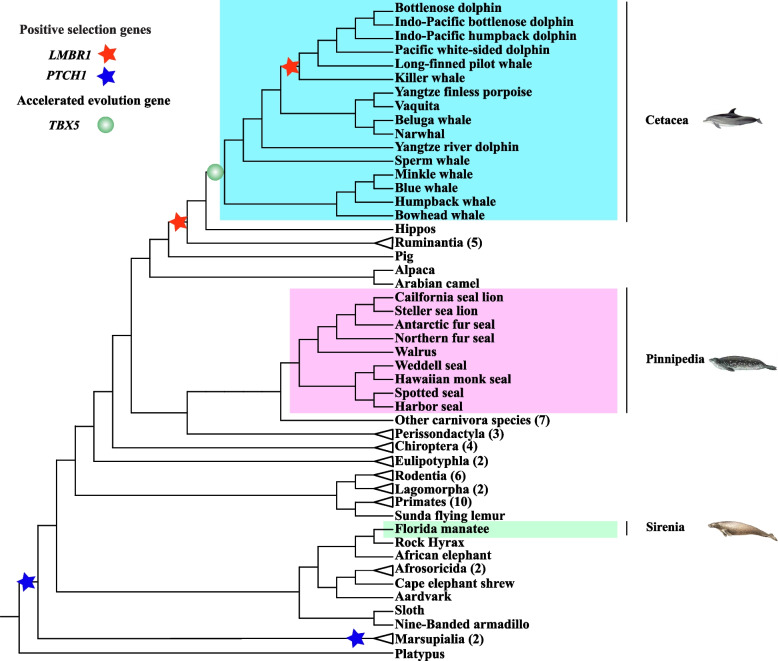
Positive selection and accelerated evolution of three limb-related genes in 80 mammals. The phylogenetic tree was from Timetree website. Sixteen representative cetacean species, eight pinnipedian species and one sirenian species were marked in light blue, pink and light green background, respectively. Positive selection in *LMBR1* and *PTCH1* were indicated in red star and blue star, respectively. Accelerated evolution of *TBX5* was shown in green ball in phylogenetic tree. Numbers in parentheses represented the number of species used in this study

One-ratio model that allows an equal ω ratio for all branches in the phylogeny showed that ω values varying from 0.0051 to 0.0864 in the 16 limb-related genes in mammals, indicating that strong functional constraint acting on these genes during mammalian evolution. To test whether positive selection restricted to some specific lineages, we compared the free-ratio model that assumes an independent ω ratio for each branch with the null one-ratio model with the same ω for all branches. The likelihood ratio test (LRT) showed that free-ratio model fitted the data better than one-ratio model at seven genes (including *LMBR1*, *BMP2*, *BMP7*, *TBX4*, *SMO, TBX5* and *PTCH1*). Evidence of positive selection was detected along the lineage to the last common ancestor (LCA) of cetruminatia and delphinidae at *LMBR1*, the LCA of marsupialia at *PTCH1*, as well as the LCA of eutheria and metatheria at *PTCH1* (Fig. 1 and Table S1). We further used the two-ratio model that allowed for different ω ratios between foreground branches and background branches to test if accelerated evolution in cetacean ancestor lineages. The result revealed that accelerated evolution was only identified in cetacean ancestor lineages at *TBX5,* whereas no such signal was found at other genes, suggesting strong selective constraint acting on the coding sequences of limb-related genes (Table 1).

**Table 1:** Two-ratio model estimating ω values in 16 limb-related genes in mammals

Gene	Models	ω0	ωc	2ΔLnL	***P***-value
*BMP2*	One-ratio (ω0)	0.0864		3.1540	0.0757
	Two-ratio (ω0, ωc)	0.0868	0.0001		
*BMP4*	One-ratio (ω0)	0.0701		0.0225	0.8808
	Two-ratio (ω0, ωc)	0.0700	0.0791		
*BMP7*	One-ratio (ω0)	0.0176		0.7374	0.3905
	Two-ratio (ω0, ωc)	0.0175	0.0387		
*FGF8*	One-ratio (ω0)	0.0051		0.4907	0.4836
	Two-ratio (ω0, ωc)	0.0051	0.0001		
*FGF10*	One-ratio (ω0)	0.0628		0.0027	0.9589
	Two-ratio (ω0, ωc)	0.0628	0.0875		
*GREM1*	One-ratio (ω0)	0.0208		0.7747	0.3789
	Two-ratio (ω0, ωc)	0.0208	0.0001		
*HAND2*	One-ratio (ω0)	0.0082		0.0004	0.9849
	Two-ratio (ω0, ωc)	0.0082	0.4476		
*LMBR1*	One-ratio (ω0)	0.0813		0.0300	0.8633
	Two-ratio (ω0, ωc)	0.0813	0.1252		
*HOXA13*	One-ratio (ω0)	0.0213		2.5987	0.1069
	Two-ratio (ω0, ωc)	0.0214	0.0001		
*PITX1*	One-ratio (ω0)	0.0142		0.8026	0.3703
	Two-ratio (ω0, ωc)	0.0143	0.0001		
*PTCH1*	One-ratio (ω0)	0.0526		0	0.9961
	Two-ratio (ω0, ωc)	0.0526	0.0526		
*SMO*	One-ratio (ω0)	0.0329		0.4960	0.4813
	Two-ratio (ω0, ωc)	0.0329	0.0007		
*SHH*	One-ratio (ω0)	0.0286		0.0514	0.8207
	Two-ratio (ω0, ωc)	0.0286	0.0001		
*TBX4*	One-ratio (ω0)	0.0381		1.5630	0.2112
	Two-ratio (ω0, ωc)	0.0379	0.1474		
*TBX5*	One-ratio (ω0)	0.0363		7.9501	0.0048
	Two-ratio (ω0, ωc)	0.0361	7.4672		
*WNT9A*	One-ratio (ω0)	0.0083		0.2138	0.6438
	Two-ratio (ω0, ωc)	0.0084	0.0001		

ωc is for the common ancestor of Cetacea (the foreground branch). ω0 is for all other branches (the background branches)

### Analysis of cis-regulatory elements of 16 limb-related genes

**Fig. 2:**
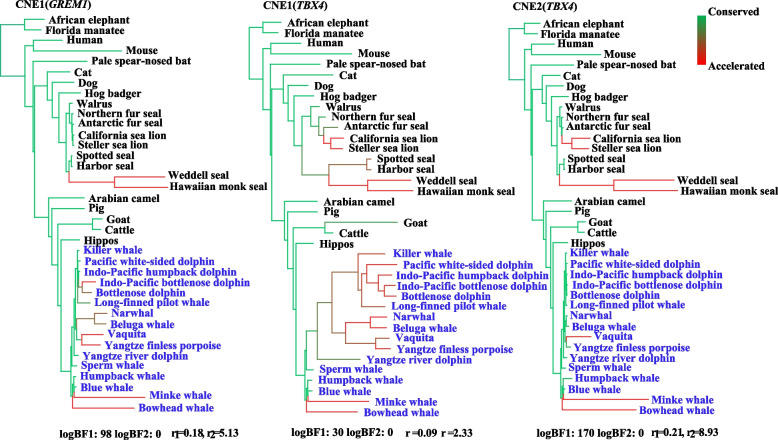
Accelerated CNEs identified in our study. Shown here are trees for three examples of accelerated CNEs. Genes targeted by CNE are shown in parentheses. Accelerated branches are marked with red and conserved branch in green. Redder branch indicates acceleration occurred at a higher rate or earlier on the branch, whereas greener one means later on the branch or no acceleration. Bayes factors (BFs) 1 & 2, the conserved (r1) and accelerated rate (r2) are listed below trees

A total of 26 CNEs (length > 50 bp) within a 1 M region of 16 limb-related genes were determined in genome alignments of 36 mammalian genomes. The result of PyloAcc displayed that a total of 10 CNEs were under accelerated evolution in cetacean lineages and other marine mammals (Fig. 2 and Fig. S1). Of them, eight CNEs were found to target flipper-related genes (*BMP7*, *BMP4*, *HAND2* and *GREM1*). For example, there were five CNEs—located near the *HAND2* gene, which was associated with anterior posterior limb mesenchyme patterning [29]—underwent rapid evolution in the linages of cetaceans and pinnipeds. One rapidly evolving CNE was separately examined in *BMP4* and *BMP7* that regulated interdigital cell death. Another one accelerated CNE was located near the *GREM1*, a known antagonist of BMPs protein [30]. By contrast, there were two identified accelerated CNEs of *TBX4* that was specific expression in hindlimb [31].

We also searched for the known enhancers of the 16 limb-related genes according to published studies and found that 17 enhancers targeting eight limb-related genes (*TBX4*, *FGF10*, *BMP4*, *FGF8*, *GREM1*, *BMP7*, *HAND2* and *SHH*) whereas no retrieved enhancers in the remaining eight genes (Table S2). For example, we retrieved six known enhancers from *FGF8*. A total of 190 cetacean-specific changes were identified in these 17 known enhancers, including 178 cetacean-specific mutations, two insertions and 10 deletions (Table S2). In addition, when we mapped these 26 CNEs to the 17 known enhancers and found all the 26 CNEs overlapped with 9 known enhancers (Table S2).

It was noted that 22 cetacean-specific changes were located in the preZRS-ZRS, a well-known enhancer of *SHH*. We further investigated potential transcription factor binding sites that might have been affected by the 22 cetacean-specific mutations in the ZRS via AnimalTFDB 3.0 (http://bioinfo.life.hust.edu.cn/AnimalTFDB#!/tfbs _predict). The result revealed 10 cetacean-specific mutations could potentially affect the 45 transcription factors binding with the ZRS (Table S3). Importantly, one cetacean-specific mutation in the ZRS A1366G was located in the homology region of 17 bp snake-specific deletions in the ZRS that occurred in HOXD13 binding sites. Furthermore, the cetacean-specific mutation A1366G was within binding sites for two known transcription factors (ETV5 and ETS1) involved in binding with the ZRS to regulate limb development [32, 33].

### Dual-luciferase reporter assay of ZRS targeting to SHH

**Fig. 3:**
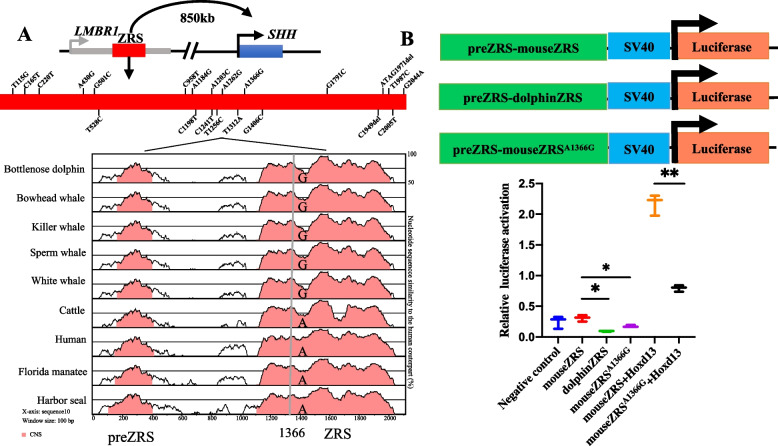
Cetacean-specific mutations in ZRS and its dual-luciferase activity. **A** cetacean-specific mutation detected in ZRS (A1336G). Schematic of the mouse *SHH* locus was shown at the top. The ZRS was located in the intron of the *LMBR1* gene (intron-exon structure not shown), 850 kb away from the promoter of *SHH*. Twenty two cetacean-specific changes were indicated by red bar. Divergence of the preZRS-ZRS sequence was shown below. **B** Luciferase reporter analyses of dolphin ZRS and its cetacean-specific mutation. One-way ANOVA was used to compare the difference between groups and data are shown as mean ± SEM (*: *P* < 0.05 and **: *P* < 0.01)

The next step was to confirm whether the cetacean-specific change in the ZRS A1366G affected the ZRS’ activities driving the expression of SHH using transfection-based dual-luciferase reporter assays. Bottlenose dolphins were the representative species in the experiment because ZRS sequences were conserved in cetaceans. We constructed three PLG3 vectors that contain ZRS sequences of bottlenose dolphin, wild-type mouse and mutated mouse with a cetacean-specific mutation (A1366G). The ZRS luciferase activity of the bottlenose dolphin was significantly lower than that of the wild-type mouse. Importantly, when we introduced cetacean-specific changes into the mouse homology ZRS (named “mouse ZRS^A1366G^”), the activity was significantly lower than that of wild-type mouse but higher than that of the dolphin ZRS. Cetacean-specific change A1366G overlapped with 17-bp snake-specific deletion in ZRS that occurred in HOXD13 binding sites [25]. Thus, to further explore whether the cetacean-specific change in ZRS affected its binding activity to transcription factor HOXD13, we co-transfected HEK293T cells with wide-type mouse ZRS or mutated mouse ZRS^A1366G^, along with the mouse HOXD13 expression vector. The result showed that the mutated mouse ZRS^A1366G^ co-transfected with HOXD13 had significantly reduced transactivation compared to the wild-type mouse (Fig. 3).

### Parallel / convergent evolution analysis among marine mammals

We examined whether the flipper forelimb showed a similar evolution pattern among different marine mammal lineages in response to aquatic environment; among the marine mammals, only one parallel / convergent site was identified: at *FGF10* (D42G). In addition, we screened 41 cetacean-specific mutations that were different from other mammals. For example, two and three specific mutations were identified in *BMP4* (I79V and H248R) and *BMP7* (Y179H, R189H and H267Q), respectively. Sixteen mutations (39.02%, 16/41) were found to be located in functional domains (Table S4).

## Discussion

### SHH signaling evolution contributed to the cetacean flipper-forelimb formation

Cetacean forelimbs evolved into flippers, reducing resistance when moving in water and facilitating the group’s movement into fully aquatic environments. It is worth noting that cetaceans are the only group of mammals with hyperphalangy. Previous studies showed that SHH signaling (such as *SHH*, *PTCH1* and *SMO*) is implicated in the maintenance of hyperphalangy involving digit elongation and additional joint induction in animal models [34]. For example, SHH protein can induce an additional phalange in the chick toe when SHH beads are planted in developing digit primordia [35], suggesting that the *SHH* gene might control the phalange number. Our study found 15 cetacean-specific amino acid changes in three genes involved in SHH signaling that might induce additional phalange formation in cetaceans. Actually, activating the SHH signaling pathway requires that *SHH* bind to its multipass transmembrane receptor Patched-1 (*PTCH1*) to transduce signals through Smoothened (*SMO*) [36]. *PTCH1* heterozygous mice were reported to display syndactyly that is similar to flipper formation [37]. Thus, cetacean specific mutations were examined in *PTCH1* (10) and *SMO* (4), which might affect the *PTCH1* and *SMO* expression pattern in forelimb development that related to the cetacean flipper forelimbs.

Earlier evidence proposed that bone morphogenetic proteins (BMPs) members, such as BMP2, BMP4, and BMP7, were involved in joint formation [38, 39]. In particularly, BMP4 expresses in the perichondrocytes appears to be necessary for normal joint formation since BMP4 application stimulates the formation of the joint-like structures in the spike of African clawed toad (*Xenopus laevis*) [40]. A total of 11 mutations were uniquely detected in cetacean lineages, nine of which located in the functional domains of BMP2 (4), BMP4 (2), and BMP7 (3), which support a potential involvement of BMP signaling in cetacean hyperphalangy that would require further investigation. BMPs were also found to promote the interdigital cell apoptosis, but its antagonism (GREM1) allowed the interdigital cells to survive in waterbirds [30]. It was previously reported that cetacean interdigital webbing occurs in the flipper-forelimb because interdigital cell apoptosis is suppressed during embryo development [4]. Our study identified nine cetacean-specific mutations in the functional domains of BMPs and one mutation in *GREM1*, which suggests that BMPs and *GREM1* take part in regulating interdigital cell survival and thus facilitate cetacean unique flipper-like forelimbs. In addition, *TBX5*, specifically expressed in the forelimb field, is required for the initiation of forelimb outgrowth, as its knockout results in complete absence of forelimbs in mice [41]. Accelerated evolution and six specific changes were found to be restricted to the *TBX5* gene in cetacean lineages. Taken together, our evidence suggests that SHH signaling (*SHH*, *PTCH1* and *SMO*), BMPs and *TBX5* might contribute to the development of the flipper-forelimb in cetaceans.

Additionally, evidence of positive selection was detected along the lineage to the last common ancestor (LCA) of marsupialia, and LCA of eutheria and metatheria at *PTCH1*. *PTCH1* has been implicated in digit loss in cow limbs in which only two digits form (3 and 4) and pig limbs that develop four digits [42, 43]. Evolution change in the number of digits is conserved across mammals, and the ancestral mammals are reported to have five digits [44]. However, a reduction of the number of digits has evolved multiple times during the evolution in mammals. For marsupial species, such as kangaroos and wallabies, the first digit is lost, although most groups also have a five-fingered phenotype. Similarly, digit loss occurred in the placental mammals, particularly in the three-toed rodents (jerboa lacks Digit I and V), an odd-toed ungulate or perissodactyl (such as single-toed horse), even-toed ungulates or artiodactyls (such as the pig with four toes and the camel with two toes) [42]. Thus, positive selection identified in *PTCH1* across ancestor lineages of marsupialia, and LCA of eutheria and metatheria might be contributed to digit reduction. Furthermore, positive selection identified in *LMBR1* along the LCA of cetaceans and ruminants (cetruminatia) might be related to their different digit number of above ancestor lineages as mutations of *LMBR1* caused polydactyly that is characterized by a digital deformity with additional digits in forelimbs and/or hindlimbs [45].

### ZRS may regulate hindlimb loss in cetaceans

The loss of the hindlimb in cetaceans facilitated the transition to a completely aquatic environment. Osteological evidence showed that the cetacean hindlimb became rudimentary because of the reduced innominate, femur and tibia [46]. Previous studies reported that the SHH protein was not detected in the posterior hindlimb bud mesenchyme of the pantropical spotted dolphin, indicating that the termination of SHH protein expression was one reason for the hindlimb loss [46]. This suggestion was corroborated in our study. We identified 22 cetacean-specific changes in preZRS-ZRS, the well-characterized *SHH* limb enhancer, that led to a significant decrease in the preZRS-ZRS activity of dolphins compared to wild-type mice. Importantly, even one cetacean-specific mutation could also reduce the ZRS activity. Similarly, only one cetacean-specific mutation in ZRS could diminish its capacity to bind a transcription factor like HOXD13. More importantly, a 17-bp deletion in ZRS proved to be the main cause of limb loss in snakes [24]. Therefore, cetacean-specific mutations in ZRS diminishing the expression of *SHH* might be the key cause of hindlimb degradation in cetaceans. Of course, changes in these regulatory sequences associated with cetacean hindlimb loss need to be further verified by transgenic mouse experiments in the future. It’s reported that HOXD (including HOXD9, HOXD10 and HOXD13), HAND2, ETV4/5 and ETS1 transcript factors can activate the ZRS in mouse limb [24, 25]. Therefore, it’s necessary to further test whether the cetacean-specific change A1366G that located in the 17-bp snake-specific deletion in ZRS can affect these protein binding sites. In addition, our observation that two CNEs were identified to be under accelerated evolution in marine mammals in *TBX4* specifically expressed in the hindlimb, and knockout of *TBX4* in zebrafish showed a pelvic fin-loss phenotype [31]. Recent research has shown one accelerated region of bats (BAR116) had a robust enhancer activity in forelimb but weak expression in hindlimb, suggesting a divergent enhancer expression pattern even in the same CNEs [47]. Therefore, we can reasonably speculate that two accelerated CNEs near *TBX4* might be related to the shortened and degraded hindlimb in marine mammals.

### *FGF10* and *GREM1* evolution may have contributed to convergent flipper-like forelimbs in marine mammals

Convergent evolution is a common phenomenon in which distantly-related species evolve similar phenotypes as they adapt to similar environmental pressures [1]. Marine mammalian groups originated from different ancestors but independently evolved convergent flipper-like limbs that can stabilize the body under the water and finish maneuvers including diving, turning and rolling to adapt to aquatic environments [48]. In our study, one parallel / convergent amino acid site was identified in marine mammals at *FGF10* that plays a key role in limb development because *FGF10*-deficient mice results in the absence of both forelimbs and hindlimbs [19]. Notably, *FGF10* has been shown to be required for induction of *FGF8* that has survival activity to inhibit interdigital cell death [19, 49]. This was demonstrated by experiments in which *FGF8* were absent from the interdigital tissues in pigs and mice, leading to freed digits, whereas presence of both *FGF8* and *GREM1* (BMP antagonist) within the interdigits of dolphins and bats was thought to maintain the survival of interdigit tissue and inhibit apoptosis [9, 50]. Interestingly, one CNE was identified to be under accelerated evolution in marine mammals at *GREM1*. Similarly, another two rapidly evolving CNEs that is located near *BMP4* and *BMP7*, which are reported to direct or indirectly regulate interdigital cell death. Thus, parallel / convergent amino acid site and accelerated CNEs identified among the different marine mammalian groups might be associated with molecular convergence in their flippers, but further functional experiments are needed to confirm this.

## Conclusions

We did evolutionary analyses of 16 limb-related genes and their cis-regulatory elements in cetaceans and compared them with those of other mammals to provide novel insights into the molecular basis of flipper forelimb and hindlimb loss in cetaceans. Our results suggest that SHH signaling (*SHH*, *PTCH1* and *SMO*) evolution contributed to cetacean flippers formation. By contrast, we suggest that low cetacean ZRS activity diminished *SHH* expression, resulting in cetacean hindlimb loss. Parallel / convergent amino acid and accelerated evolution of CNEs in marine mammalian lineages in *FGF10* and *GREM1*, respectively, might be associated with convergent flipper phenotypes among marine mammals.

## Methods

### Sequence retrieval and alignment

We used a total of 26 marine mammalian species (including 16 cetacean species, nine pinnipeds and one species of manatee) and 54 terrestrial relatives (Table S5). Sixteen limb-related gene sequences were first retrieved from the Ensembl public database (http://www.ensembl.org/index.html), NCBI (https://www.ncbi.nlm.nih.gov/) and OrthoMaM database (https://orthomam2.mbb.univ-monto2.fr:8080/OrthoMaM_v10b9/); *Phoca largha* and *Arctonyx collaris* sequences came from our unpublished data. Then, the coding sequences (CDS) of each gene were aligned in Fasparser 2.0 (https://github.com/Sun-Yanbo/FasParser) using Prank [51] and nonhomologous fragments were deleted by Gblocks [52] with the parameters “-t = codons, -b5 = half”. To verify the accuracy of these sequences from published datasets, we amplified *FGF8* genes in eight species of odontocetes and one mysticete species (Table S6). We used BLAST to extend each exon sequence in known cetacean genomes and compared them with the full-length coding sequences to design primers using PrimerPrimer5 [53] for each exon in the *FGF8* gene (Table S7).

### Analysis of selection pressure

We estimated the ratio (ω) of nonsynonymous (*d*_N_) to synonymous (*d*_S_) codon substitution based on maximum likelihood models using the CODEML program from PAML 4.7 software [54]. The ω ratio (ω = *d*_N_*/d*_S_) indicates the change in selective pressures, while ω > 1, ω < 1 and ω =1 indicates positive selection, purifying selection and neutral evolution, respectively. A well-supported phylogeny of mammals from the TimeTree website [55] was used as the input tree in our analyses. To evaluate whether accelerated evolution and positive selection were limited to specific lineages, the branch models including two-ratio and free-ratio model were performed in the all-mammals dataset. The two-ratio model allowed for different ω ratios between foreground branches and background branches, whereas the free-ratio model assumed that each branch has an independent ω ratio [54]. Results of both the two-ratio and free-ratio models were compared with the null model—a one-ratio model that allowed an equal ω ratio for all branches in the phylogeny. When the value of *d*_N_ or *d*_S_ in each ω value is less than 0.0002, we considered it as an outlier “n/a” [56]. The relative goodness-of-fit of the nested models was determined using a likelihood ratio test (LRT) that was performed by comparing twice the difference in ln likelihood scores (2ΔlnL) against a χ^2^ distribution, with the number of degrees of freedom corresponding to the difference in number of parameters between the nested models. All nested models were statistically different from the null model (*P* < 0.05).

### Evolution analysis of regulatory elements in 16 limb-related genes

To identify potential regulatory elements of the 16 limb-related genes, we selected 38 species (including 26 marine mammals and 12 terrestrial mammals) with high-quality genomes and generated the sequence alignment using LASTZ with the parameters K = 2400, L = 3000, Y = 9400, H = 2000 [57] using the human genome (GRCh38/hg38) as a reference. The MULTIZ 11.2 program [58] was used to build multiple sequence alignments. Based on previous studies, we scanned the CNEs within approximately 1 Mb candidate region of 16 limb-related genes using PhastCons (http://compgen.cshl.edu/phast/phastCons-HOWTO.html), which required us to estimate neutral branch lengths by PhyloFit. To estimate whether cetacean-accelerated CNEs were present along cetacean linages, we used PhyloAcc software [59]. Bayes factors (BF) with BF1 > 20 and BF2 > 0 for elements were considered to have strong evidence of cetacean-specific acceleration.

We also retrieve the known enhancers targeting the 16 limb-related genes according to published studies, and then identified cetacean-specific changes in enhancers using Fasparser 2.0 [60].

### Dual-luciferase reporter assay

To explore whether the cetacean-specific mutations in enhancers effect the regulative activity of the target genes, we selected ZRS, an enhancer of the *SHH* gene, to further identify the activation ability by Dual-Luciferase Reporter Assay System. ZRS sequences of a representative cetacean species—bottlenose dolphin—and the control group of mice were amplified from muscle tissue. Then, the two sequences were cloned into a pGL3-SV40 firefly luciferase reporter vector (E1771, Promega), including pGL3-dZRS-Luc and pGL3-mZRS-Luc. Site-directed mutagenesis to construct pGL3-mZRSmut-Luc was performed by introducing the cetacean-specific mutation A1366G with the QuickMutation™ Site-Directed Mutagenesis Kit (D0206, Beyotime) using the pGL3-mZRS-Luc vector as the template. The transcription factor gene *HOXD13* was amplified from mouse embryo cDNA and then cloned into a pcDNA3.1^+^ plasmid from the GenScript Company (Jiangsu, China). Primers related to constructs are shown in Table S7.

HEK293T cells (ATCC; #CRL-11268) were plated in 24 well plates in Dulbecco’s Modified Eagle’s Medium (WISENT) + 10% fetal bovine serum (Gibco; #16140–071) and grown night. The cells were transfected with *HOXD13* expression vectors, firefly luciferase and renilla luciferease plasmids by lipofectamine 293™ (SL100668, SignaGen). The Dual-Luciferase® Reporter (DLRTM) Assay System (DL101–01, Vazyme) was used to produce nuclear lysates and luminescence reactions from firefly and renilla luciferases. Quantifications were performed using at least three independent experimental groups and one-way ANOVA was used to determine significance.

In addition, to identify transcription factor binding site (TFBS) changes in the ZRS, we used AnimalTFDB v3.0 (http://bioinfo.life.hust.edu.cn/AnimalTFDB/) to predict changes in TFBSs caused by 22 cetacean-specific changes in ZRS. We called a TFBS a hit if its corrected *p*-value less than the threshold of 0.05 [61].

### Convergent analysis among marine mammals

We used Fasparser 2.0 [60] to identify parallel / convergent sites in 16 limb-related genes among three marine groups. Fasparser 2.0 was further used to screen the cetacean-specific changes in coding genes and CNEs. We detected parallel / convergent / specific sites located in protein functional domains using PFAM (http://pfam.xfam.org).

## Supplementary Information

**Additional file 1: Table S1.** Selective pressure analyses of 16 limb-related genes and evidence of positive selection on the *LMBR1* and *PTCH1* genes in mammals using branch models. **Table S2.** Cetacean-specific site mutations number in the 17 known enhancers of limb-related genes and genomic position of overlapped CNEs. **Table S3.** ZRS transcription factor binding sites analysis. **Table S4.** Cetacean-specific changes identified in 16 limb-related genes. **Table S5.** Ensembl transcript IDs, GenBank accession numbers of limb-related genes used in our study. **Table S6.** *FGF*8 gene amplification in this study. **Table S7.** Primers used in this study.

**Additional file 2: Fig. S1.** Accelerated CNEs identified in our study.

## Data Availability

The data generated and analyzed during this study are included in this article and its additional files, including 8 tables and 4 figures.

## Abbreviations

CNEs: Conserved Noncoding Elements
*WNT9A*: Wnt family member 9a
*SHH*: Sonic hedgehog
AER: Apical Ectodermal Ridge
FGFs: Fibroblast Growth Factors
*PITX1*: Paired-like homeodomain 1
ZRS: ZPA Regulation Sequence
ZPA: Zone of Polarizing Activity
HLEB: Hindlimb enhancer B
LCA: Last Common Ancestor
*PTCH1*: Patched-1
*SMO*: Smoothened
CDS: Coding sequences
BLAST: basic local alignment search tool
LRT: likelihood ratio test
BF: Bayes Factors
PAML: phylogenetic analysis by maximum likelihood
