# Supplementary material for: Evolutionary genetics of flipper forelimb and hindlimb loss from limb development-related genes in cetaceans
Source: BMC Genomics. 2022 Dec 2;23:797. doi: 10.1186/s12864-022-09024-3 (PMC9719152; doi:10.1186/s12864-022-09024-3)
Supplement: Supplementary file 1 — Additional file 1: Table S1. Selective pressure analyses of 16 limb-related genes and evidence of positive selection on the LMBR1 and PTCH1 genes in mammals using branch models. Table S2. Cetacean-specific site mutations number in the 17 known enhancers of limb-related genes and genomic position of overlapped CNEs. Table S3. ZRS transcription factor binding sites analysis. Table S4. Cetacean-specific changes identified in 16 limb-related genes. Table S5. Ensembl transcript IDs, GenBank accession numbers of limb-related genes used in our study. Table S6. FGF8 gene amplification in this study. Table S7. Primers used in this study. [file 12864_2022_9024_MOESM1_ESM.doc]

**Evolutionary genetics of** **flipper forelimb and hindlimb loss from limb** **development-related genes in cetaceans**

Linxia Sun**,** Xinghua Rong, Xing Liu, Zhenpeng Yu, Qian Zhang, Wenhua Ren, Guang Yang*, Shixia Xu*

Jiangsu Key Laboratory for Biodiversity and Biotechnology, College of Life Sciences, Nanjing Normal University, Nanjing 210023, China

* Corresponding to:

Shixia Xu, E-mail: xushixia@njnu.edu.cn (SX).

Guang Yang, E-mail: gyang@njnu.edu.cn (GY).

**Additional files 1**

**Table S1. Selective pressure analyses of 16 limb-related genes and evidence of positive selection on the *LMBR1* and *PTCH1* genes in mammals using branch models.**

| **Gene** | **Models** | **-lnL^a^** | **Models compared** | **2ΔLnL^b^** | **d.f.** | ***P*-value** | **Parameter estimates** |  |
| --- | --- | --- | --- | --- | --- | --- | --- | --- |
| *LMBR1* | Branch (last common ancestral branch of Cetruminatia) | | | | | |  | |
|  | M0: one-ratio | 15395.288 |  |  |  |  | ω=0.0813, *d*N=0.6379, *d*S=7.8458 |  |
|  | M1: free-ratio | 15564.105 | M1 versus M0 | 337.6353 | 153 | <0.001 | ω=1.2256, *d*N=0.0032, *d*S=0.0026 |  |
|  | Branch (last common ancestral branch of Delphinidae) | | | | | |  | |
|  | M0: one-ratio | 15395.288 |  |  |  |  | ω=0.0813, *d*N=0.6379, *d*S=7.8458 |  |
|  | M1: free-ratio | 15564.105 | M1 versus M0 | 337.6353 | 153 | <0.001 | ω=1.2522, *d*N=0.0029, *d*S=0.0024 |  |
| *PTCH1* | Branch (last common ancestral branch of Marsupialia) | | | | | |  | |
|  | M0: one-ratio | 44696.059 |  |  |  |  | ω=0.0528, *d*N=0.5775, *d*S=10.9841 |  |
|  | M1: free-ratio | 44907.328 | M1 versus M0 | 422.5367 | 155 | <0.001 | ω=6.7222, *d*N=0.0182, *d*S=0.0027 |  |
|  | Branch (last common ancestral branch of Eutheria and Metatheria) | | | | | |  | |
|  | M0: one-ratio | 44696.059 |  |  |  |  | ω=0.0528, *d*N=0.5775, *d*S=10.9841 |  |
|  | M1: free-ratio | 44907.328 | M1 versus M0 | 422.5367 | 155 | <0.001 | ω=1.8827, *d*N=0.0413, *d*S=0.022 |  |

Note: a, lnL is the log-likelihood score; b, twice the difference in ln L between the two models compared.

**Table S2**. Cetacean-specific site mutations number in the 17 known enhancers of limb-related genes and genomic position of overlapped CNEs.

| **Gene** | **Enhancer** | **Genomic position^*^** | **Cetacean-specific sites** | **Insertions** | **Deletions** | **Genomic position of overlapped CNEs** |
| --- | --- | --- | --- | --- | --- | --- |
| *TBX4* | e1 | chr17:61443108-61444201 | 23 |  | 6 |  |
|  | e2 | chr17:61532022-61535334 | 23 | 1 | 1 | chr17:61532108-61532195; chr17:61532220-61532578; chr17:61532877-61533025; chr17:61533033-61533107^a^; chr17:61533126-61533312; chr17:61534110-61534288^a^; chr17:61534353-61534878; chr17:61535273-61535375 |
| *FGF10* | e3 | chr5:44390455-45062032 | 11 |  |  |  |
| *BMP4* | e4 | chr14:53998926-54000072 | 7 |  |  | chr14:53999078-53999362^a^ |
| *FGF8* | e5 | chr10:101506374-101508221 | 15 |  |  |  |
|  | e6 | chr10:101612567-101616153 | 24 |  |  |  |
|  | e7 | chr10:101617274-101618364 | 9 |  |  |  |
|  | e8 | chr10:101643380-101644568 | 7 |  |  |  |
|  | e9 | chr10:101660069-101660689 | 1 |  |  |  |
|  | e10 | chr10:101671689-101768952 | 0 |  |  | chr10:101671825-101672065; chr10:101672090-101672144 |
| *GREM1* | e11 | chr15:32851115-32851565 | 3 |  |  | chr15:32851266-32851492^a^ |
|  | e12 | chr15:32798236-32798743 | 3 |  |  | chr15:32798250-32798610 |
| *BMP7* | e13 | chr20:57259203-57259684 | 2 |  |  | chr20:57258040-57258139^a^; chr20:57258434-57258507; chr20:57259151-57259690 |
| *HAND2* | e14 | chr4:174036285-174038798 | 13 | 1 | 1 | chr4:174036681-174036835; chr4:174037096-174037234^a^; chr4:174037249-174037662^a^; chr4:174037674-174037738; chr4:174037746-174037815; chr4:174037829-174037971^a^ |
|  | e15 | chr4:173943983-173946130 | 15 |  |  | chr4:173944981-173945102^a^; chr4:173945329-173945407; chr4:173945459-173945635^a^ |
|  | e16 | chr4:173878641-173880679 | 2 |  |  | chr4:173879384-173879757 |
| *SHH* | e17 | chr7:156791088-156793079 | 20 |  | 2 |  |

Note: ^*^, human genome (GRCh38/hg38) as a reference; ^a^, CNEs under accelerated evolution.

**Table S3.** ZRS transcription factor binding sites analysis.

| **Cetacean-specific changes** | **Transcription factor** | **Start** | **Stop** | **P-value** | **Transcription factor binding sites** |
| --- | --- | --- | --- | --- | --- |
| C165T | NCOR2 | 163 | 174 | 0.046 | CCCTCCTGGGGT |
| C220T | MYC | 209 | 224 | 0.0339 | GTGGCACATGGCTCCC |
|  | MAX | 210 | 221 | 0.0133 | TGGCACATGGCT |
|  | MYC | 211 | 222 | 0.024 | GGCACATGGCTC |
|  | MAX | 211 | 222 | 0.0411 | GGCACATGGCTC |
|  | MYC | 212 | 223 | 0.016 | GCACATGGCTCC |
| G501C | FOXM1 | 483 | 502 | 0.0249 | CACTTTTTTTTTTTAAATGA |
|  | TRIM28 | 487 | 508 | 0.0207 | TTTTTTTTTTAAATGAATTCTC |
| C958T | USF1 | 951 | 975 | 0.0335 | TACTGGCCAGTGTTAAATGAGAGTA |
| T1256C, A1262G | SP2 | 1256 | 1270 | 0.0168 | TCCTAGAGTGTCCAG |
| A1366G | THRA | 1359 | 1368 | 0.027 | CTGAGGTCAC |
|  | PML | 1359 | 1367 | 0.0139 | CTGAGGTCA |
|  | SPI1 | 1360 | 1378 | 0.00998 | TGAGGTCACTTCCTCTCTT |
|  | RXRB | 1360 | 1369 | 0.0312 | TGAGGTCACT |
|  | SMARCA4 | 1362 | 1377 | 0.00998 | AGGTCACTTCCTCTCT |
|  | SPI1 | 1362 | 1379 | 0.028 | AGGTCACTTCCTCTCTTA |
|  | SPI1 | 1362 | 1373 | 0.0375 | AGGTCACTTCCT |
|  | MED1 | 1362 | 1377 | 0.0451 | AGGTCACTTCCTCTCT |
|  | SPI1 | 1363 | 1378 | 0.00126 | GGTCACTTCCTCTCTT |
|  | SPI1 | 1363 | 1379 | 0.0017 | GGTCACTTCCTCTCTTA |
|  | SPIB | 1363 | 1378 | 0.0019 | GGTCACTTCCTCTCTT |
|  | SPI1 | 1363 | 1380 | 0.0021 | GGTCACTTCCTCTCTTAA |
|  | STAT1 | 1363 | 1378 | 0.00584 | GGTCACTTCCTCTCTT |
|  | FLI1 | 1363 | 1378 | 0.0133 | GGTCACTTCCTCTCTT |
|  | MYC | 1363 | 1377 | 0.0114 | GGTCACTTCCTCTCT |
|  | SPI1 | 1363 | 1374 | 0.0182 | GGTCACTTCCTC |
|  | EP300 | 1363 | 1378 | 0.0377 | GGTCACTTCCTCTCTT |
|  | SPIB | 1364 | 1379 | 0.000723 | GTCACTTCCTCTCTTA |
|  | FLI1 | 1364 | 1378 | 0.013 | GTCACTTCCTCTCTT |
|  | ETV5 | 1364 | 1375 | 0.0165 | GTCACTTCCTCT |
|  | RUNX1 | 1364 | 1375 | 0.0193 | GTCACTTCCTCT |
|  | ERG | 1364 | 1379 | 0.037 | GTCACTTCCTCTCTTA |
|  | RBPJ | 1364 | 1375 | 0.0399 | GTCACTTCCTCT |
|  | CBFB | 1364 | 1378 | 0.0424 | GTCACTTCCTCTCTT |
|  | ETS1 | 1364 | 1376 | 0.0433 | GTCACTTCCTCTC |
|  | CREBBP | 1364 | 1375 | 0.0449 | GTCACTTCCTCT |
|  | ELF1 | 1364 | 1377 | 0.0378 | GTCACTTCCTCTCT |
|  | SPI1 | 1365 | 1379 | 0.000561 | TCACTTCCTCTCTTA |
|  | CREB1 | 1365 | 1376 | 0.000769 | TCACTTCCTCTC |
|  | MYB | 1365 | 1376 | 0.000962 | TCACTTCCTCTC |
|  | SPI1 | 1365 | 1380 | 0.00108 | TCACTTCCTCTCTTAA |
|  | RXRG | 1365 | 1376 | 0.00195 | TCACTTCCTCTC |
|  | RXRA | 1365 | 1376 | 0.00198 | TCACTTCCTCTC |
|  | TCF3 | 1365 | 1376 | 0.00257 | TCACTTCCTCTC |
|  | PML | 1365 | 1376 | 0.00267 | TCACTTCCTCTC |
|  | STAT3 | 1365 | 1376 | 0.00271 | TCACTTCCTCTC |
|  | RUNX1 | 1365 | 1379 | 0.00596 | TCACTTCCTCTCTTA |
|  | RUNX1 | 1365 | 1376 | 0.00814 | TCACTTCCTCTC |
|  | NCOR2 | 1365 | 1376 | 0.0145 | TCACTTCCTCTC |
|  | CREBBP | 1365 | 1376 | 0.0178 | TCACTTCCTCTC |
|  | ETS1 | 1365 | 1377 | 0.0222 | TCACTTCCTCTCT |
|  | ETS1 | 1365 | 1376 | 0.0349 | TCACTTCCTCTC |
|  | MYC | 1365 | 1376 | 0.0483 | TCACTTCCTCTC |
|  | EP300 | 1365 | 1377 | 0.0497 | TCACTTCCTCTCT |
|  | ERG | 1365 | 1376 | 0.0494 | TCACTTCCTCTC |
|  | BRD4 | 1366 | 1377 | 0.000511 | CACTTCCTCTCT |
|  | SPI1 | 1366 | 1379 | 0.000781 | CACTTCCTCTCTTA |
|  | BCL6 | 1366 | 1377 | 0.000843 | CACTTCCTCTCT |
|  | NCOR2 | 1366 | 1377 | 0.00104 | CACTTCCTCTCT |
|  | IRF4 | 1366 | 1377 | 0.0037 | CACTTCCTCTCT |
|  | SPI1 | 1366 | 1376 | 0.00403 | CACTTCCTCTC |
|  | HDAC1 | 1366 | 1377 | 0.00645 | CACTTCCTCTCT |
|  | IRF4 | 1366 | 1377 | 0.009 | CACTTCCTCTCT |
|  | MYC | 1366 | 1377 | 0.0244 | CACTTCCTCTCT |
|  | CDK8 | 1366 | 1374 | 0.0138 | CACTTCCTC |
|  | STAT5A | 1366 | 1374 | 0.0196 | CACTTCCTC |
|  | YY1 | 1366 | 1374 | 0.0404 | CACTTCCTC |
| G1406C | SPI1 | 1391 | 1406 | 0.0187 | TGACCAGGTGGAGGCG |
|  | CTCF | 1391 | 1406 | 0.0493 | TGACCAGGTGGAGGCG |
|  | MYC | 1392 | 1406 | 0.0266 | GACCAGGTGGAGGCG |
|  | RNF2 | 1394 | 1409 | 0.0141 | CCAGGTGGAGGCGAAG |
|  | MAX | 1394 | 1409 | 0.0405 | CCAGGTGGAGGCGAAG |
| C2005T | EZH2 | 1993 | 2008 | 0.014 | CTCTCTGCTCTCCCCC |
|  | CTCF | 1997 | 2021 | 0.0174 | CTGCTCTCCCCCACTAGGGAGCACT |
|  | SPI1 | 1999 | 2023 | 0.0445 | GCTCTCCCCCACTAGGGAGCACTGG |
|  | CTCF | 2001 | 2020 | 0.0272 | TCTCCCCCACTAGGGAGCAC |
|  | SPI1 | 2001 | 2022 | 0.0494 | TCTCCCCCACTAGGGAGCACTG |
|  | CTCF | 2002 | 2022 | 0.0167 | CTCCCCCACTAGGGAGCACTG |
|  | CTCF | 2003 | 2022 | 0.0318 | TCCCCCACTAGGGAGCACTG |
|  | TBL1X | 2004 | 2025 | 0.00882 | CCCCCACTAGGGAGCACTGGTT |
|  | SPI1 | 2004 | 2019 | 0.014 | CCCCCACTAGGGAGCA |
|  | CBFB | 2004 | 2028 | 0.0174 | CCCCCACTAGGGAGCACTGGTTTCT |
|  | SPI1 | 2004 | 2028 | 0.0176 | CCCCCACTAGGGAGCACTGGTTTCT |
|  | CTCF | 2004 | 2022 | 0.00988 | CCCCCACTAGGGAGCACTG |
|  | CTCF | 2004 | 2021 | 0.0211 | CCCCCACTAGGGAGCACT |
|  | CTCF | 2004 | 2020 | 0.0306 | CCCCCACTAGGGAGCAC |
|  | SPI1 | 2004 | 2025 | 0.029 | CCCCCACTAGGGAGCACTGGTT |
|  | TRIM28 | 2004 | 2019 | 0.02 | CCCCCACTAGGGAGCA |
|  | CTCF | 2004 | 2025 | 0.0239 | CCCCCACTAGGGAGCACTGGTT |
|  | ZNF384 | 2004 | 2025 | 0.0241 | CCCCCACTAGGGAGCACTGGTT |
|  | CTCF | 2004 | 2019 | 0.0295 | CCCCCACTAGGGAGCA |
|  | STAG1 | 2004 | 2019 | 0.0367 | CCCCCACTAGGGAGCA |
|  | SPI1 | 2005 | 2020 | 0.00793 | CCCCACTAGGGAGCAC |
|  | CTCF | 2005 | 2023 | 0.0159 | CCCCACTAGGGAGCACTGG |
|  | MYC | 2005 | 2020 | 0.0193 | CCCCACTAGGGAGCAC |
|  | SPI1 | 2005 | 2028 | 0.0217 | CCCCACTAGGGAGCACTGGTTTCT |
|  | SPI1 | 2005 | 2029 | 0.0165 | CCCCACTAGGGAGCACTGGTTTCTT |
|  | CTCF | 2005 | 2024 | 0.0195 | CCCCACTAGGGAGCACTGGT |
|  | CTCF | 2005 | 2025 | 0.0274 | CCCCACTAGGGAGCACTGGTT |
|  | CTCF | 2005 | 2022 | 0.0206 | CCCCACTAGGGAGCACTG |
|  | CTCF | 2005 | 2020 | 0.0421 | CCCCACTAGGGAGCAC |
|  | MYC | 2005 | 2019 | 0.0266 | CCCCACTAGGGAGCA |
|  | CTCF | 2005 | 2019 | 0.046 | CCCCACTAGGGAGCA |
| G2044A | MEF2A | 2029 | 2044 | 0.0364 | TTCCAGAAATAGTAAG |

Note: The shaded areas indicate mouse nucleotide sites different from cetacean-specific changes.

**Table S4.** Cetacean-specific changes identified in 16 limb-related genes.

| **Gene** | **AA positions** | **AA changes**^a^ |
| --- | --- | --- |
| *PTCH1* | 14 | Glu-Asp |
|  | 577 | Thr-Ser |
|  | 766 | Ser-Asn |
|  | 1058 | Val-Leu |
|  | 1100 | Ala-Ser |
|  | 1125 | His-Gln |
|  | 1159 | Gly-Cys |
|  | 1193 | Ala-Cys |
|  | 1249 | Cys-Arg |
|  | 1279 | Ser-Thr |
| *SMO* | 97 | Asp-Asn |
|  | 632 | Lys-Arg |
|  | 647 | Pro-Ser |
|  | 686 | Cys-Tyr |
| *GREM1* | 68 | Met-Val |
| *BMP4* | 79 | Ile-Val |
|  | 248 | His-Arg |
| *BMP2* | 20 | Gly-Val |
|  | 68 | Asp-Gly |
|  | 194 | Asn-Thr |
|  | 229 | Leu-Pro |
|  | 231 | Glu-Asp |
|  | 256 | Ile-Arg |
| *TBX4* | 382 | Ala-Thr |
|  | 417 | Met-Val |
|  | 444 | Pro-Gln |
|  | 451 | Asn-Ser |
|  | 480 | Leu-Phe |
| *SHH* | 35 | His-Asn |
| *TBX5* | 383 | Ala-Val |
|  | 399 | Thr-Ala |
|  | 404 | Pro-Ser |
|  | 460 | Val-Met |
|  | 513 | Ser-Asn |
|  | 515 | Asn-Ser |
| *LMBR1* | 260 | Tyr-His |
|  | 270 | Arg-Asp |
|  | 441 | Val-Met |
| *BMP7* | 179 | Tyr-His |
|  | 189 | Arg-His |
|  | 267 | His-Gln |

Note: ^a^, the right of ‘-’ is cetacean specific AA changes.

**Table S5.** Ensembl transcript IDs, GenBank accession numbers of limb-related genes used in our study

| **Order** | **Scientific name** | **English name** | **Sequence origin** |
| --- | --- | --- | --- |
| **Cetartiodactyla** |  |  |  |
|  | *Lagenorhynchus Obliquidens* | Pacific White-Sided Dolphin | NCBI (GCA_003676395.1) |
|  | *Tursiops Truncatus* | Bottlenose Dolphin | OrthoMaM |
|  | *Lipotes Vexilifer* | Yangtze River Dolphin | OrthoMaM |
|  | *Orcinus Orca* | Killer Whale | OrthoMaM |
|  | *Delphinapterus Leucas* | Beluga Whale | OrthoMaM |
|  | *Physeter Catodon* | Sperm Whale | NCBI (GCA_002837175.2) |
|  | *Globicephala Melas* | Long-Finned Pilot Whale | NCBI (GCA_006547405.1) |
|  | *Monodon Monoceros* | Narwhal | NCBI (GCA_005190385.2) |
|  | *Balaena mysticetus* | Bowhead whale | Unpublished |
|  | *Balaenoptera musculus* | Blue Whale | NCBI([GCA_009873245.3](https://ftp.ncbi.nlm.nih.gov/genomes/genbank/vertebrate_mammalian/Balaenoptera_musculus/latest_assembly_versions/GCA_009873245.3_mBalMus1.pri.v3/)) |
|  | *Megaptera novaeangliae* | Humpback whale | NCBI (GCA_004329385.1) |
|  | *Neophocaena phocaenoides* | Finless Porpoise | unpublished |
|  | *Phocoena sinus* | Vaquita | NCBI(GCA_008692025.1) |
|  | *Sousa chinensis* | Indo-Pacific humpback dolphin | NCBI(GCA_007760645.1) |
|  | *Tursiops aduncus* | Indo-Pacific bottlenose dolphin | NCBI(GCA_003227395.1) |
|  | *Balaenoptera Acutorostrata* | Minke Whale | OrthoMaM |
|  | *Bubalus Bubalis* | Water Buffalo | OrthoMaM |
|  | *Bos Taurus* | Cattle | OrthoMaM |
|  | *Bos Indicus* | Zebu | OrthoMaM |
|  | *Capra Hircus* | Goat | OrthoMaM |
|  | *Ovis Aries* | Sheep | OrthoMaM |
|  | *Sus Scrofa* | Pig | OrthoMaM |
|  | *Camelus Dromedarius* | Arabian camel | OrthoMaM |
|  | *Hippopotamus amphibius* | Hippos | NCBI (GCA_004027065.2) |
|  | *Vicugna pacos* | Alpaca | NCBI(GCA_000164845.4) |
| **Perissodactyla** |  |  |  |
|  | *Ceratotherium simum simum* | Southern White Rhinoceros | OrthoMaM |
|  | *Eqquus Caballus* | Horse | OrthoMaM |
|  | *Equus Asinus* | Ass | OrthoMaM |
| **Carnivora** |  |  |  |
|  | *Neomonachus Schauinslandi* | Hawaiian Monk Seal | OrthoMaM |
|  | *Leptonychotes Weddellii* | Weddell Seal | OrthoMaM |
|  | *Zalophus Californianus* | California Sea Lions | NCBI (GCA_900631625.1) |
|  | *Callorihinus Ursinus* | Northern Fur Seals | NCBI (GCA_003265705.1) |
|  | *Eumetopioas Jubatus* | Steller Sea Lion | NCBI (GCA_004028035.1 |
|  | *Odobenus Rosmarus* | Walrus | OrthoMaM |
|  | *Ursus Maritimus* | Polar Bear | OrthoMaM |
|  | *Acinonyx Jubatus* | Cheetah | OrthoMaM |
|  | *Felis Catus* | Cat | OrthoMaM |
|  | *Ailuropoda Melanoleuca* | Giant Panda | OrthoMaM |
|  | *Canis Familiaris* | Dog | OrthoMaM |
|  | *Arctonyx collaris* | Hog Badger | unpublished |
|  | *Arctocephalus gazella* | Antarctic Fur seal | NCBI (GCA_900642305.1) |
|  | *Phoca largha* | Spotted seal | unpublished |
|  | *Phoca vitulina* | Harbour seal | NCBI (GCA_004348235.1) |
|  | *Ursus arctos* | Brown Bear | NCBI (GCA_003584765.1) |
| **Pholidota** |  |  |  |
|  | *Manis Javanica* | Sunda Pangolin | NCBI (GCA_001685135.1) |
| **Chiroptera** |  |  |  |
|  | *Rousettus Aegyptiacus* | Egyptian Fruit Bat | OrthoMaM |
|  | *Hipposideros Armiger* | Great Himalayan Leaf-Nosed Bat | OrthoMaM |
|  | *Phyllostomus Discolor* | Pale Spear-Nosed Bat | NCBI (GCA_004126475.2) |
|  | *Myotis lucifugus* | Little Brown Bat | NCBI (GCA_000147115.1) |
|  | *Pteropus vampyrus* | Malayan Flying Fox | NCBI (GCA_000151845.2) |
| **Rodentia** |  |  |  |
|  | *Nanospalax Galili* | Mole Rat | OrthoMaM |
|  | *Microtus Ochrogaster* | Prairie Vole | OrthoMaM |
|  | *Mus Pahari* | Gairdner'S Shrew-Mouse | OrthoMaM |
|  | *Mus Musculus* | Mouse | OrthoMaM |
|  | *Mus Caroli* | Ryukyu Mouse | OrthoMaM |
|  | *Rattus Norvegicus* | Brown Rat | OrthoMaM |
| **Lagomorpha** |  |  |  |
|  | *Oryctolagus Cuniculus* | Rabbit | OrthoMaM |
|  | *Ochotona Princeps* | American Pika | OrthoMaM |
| **Dermoptera** |  |  |  |
|  | *Galeopterus Variegatus* | Sunda Flying Lemur | OrthoMaM |
| **Scandentia** |  |  |  |
|  | *Tupaia Belangeri* | Northern Treeshrew | OrthoMaM |
| **Primates** |  |  |  |
|  | *Macaca Mulatta* | Rhesus Macaque | OrthoMaM |
|  | *Chlorocebus Sabeaus* | Green Monkey | OrthoMaM |
|  | *Piliocolobus Tephrosceles* | Ugandan Red Colobus | OrthoMaM |
|  | *Pan Troglodytes* | Chimpanzee | OrthoMaM |
|  | *Homo Sapiens* | Human | OrthoMaM |
|  | *Cebus Capucinus* | White-Headed Capuchin | OrthoMaM |
|  | *Microcebus Murinus* | Gray Mouse Lemur | OrthoMaM |
|  | *Propithecus Coquereli* | Coquerel'S Sifaka | NCBI (GCA_000956105.1) |
|  | *Callithrix jacchus* | White-tufted-ear Marmoset | NCBI (GCA_009663435.2) |
| **Edentata** |  |  |  |
|  | *Choloepus hoffmanni* | Sloth | NCBI (GCA_000164785.2) |
|  | *Dasypus Novemcinctus* | Nine-Banded Armadillo | OrthoMaM |
| **Proboscidea** |  |  |  |
|  | *Loxodonta Africanna* | African Elephant | OrthoMaM |
| **Sirenia** |  |  |  |
|  | *Trichechus Manatus Latirostris* | Florida Manatee | OrthoMaM |
| **Hyracoidea** |  |  |  |
|  | *Procavia Capensis* | Rock Hyrax | OrthoMaM |
| **Tubulidentata** |  |  |  |
|  | *Orycteropus Afer* | Aardvark | OrthoMaM |
| **Monotrysia** |  |  |  |
|  | *Ornithorhynchus Anatinus* | Platypus | NCBI (GCA_004115215.2) |
| **Marsupialia** |  |  |  |
|  | *Sarcophilus harrisii* | Tasmanian Devil | OrthoMaM |
|  | *Monodelphis Domestica* | Gray Short-tailed Opossum | OrthoMaM |
| **Macroscelidea** |  |  |  |
|  | *Elephantulus Edwardii* | Cape Elephant Shrew | OrthoMaM |
| **Eulipotyphla** |  |  |  |
|  | *Condylura cristata* | Star-Nosed Mole | OrthoMaM |
|  | *Erinaceus Europaeus* | Hedgehog | OrthoMaM |
| **Afrosoricida** |  |  |  |
|  | *Chrysochloris Asiatica* | Cape Golden Mole | OrthoMaM |
|  | *Echinops Telfairi* | Lesser Hedgehog Tenrec | OrthoMaM |

**Table S6.** *FGF*8 gene amplification in this study.

| **Species** | **Latin name** | **Length (bp)** | | **Coverage** |
| --- | --- | --- | --- | --- |
| Dwarf sperm whale | *Kogia sima* | 735 | 100% | |
| Indo-Pacific bottlenose dolphin | *Tursiops aduncus* | 666 | 90.6% | |
| Indo-Pacific humpback dolphin | *Sousa chinensis* | 735 | 100% | |
| Common dolphin | *Delphinus delphis* | 735 | 100% | |
| Beluga whale | *Delphinapterus leucas* | 735 | 100% | |
| Grampus griseus | *Grampus griseus* | 735 | 100% | |
| Blainville’s beaked whale | *Mesoplodon densirostris* | 735 | 100% | |
| Omura’s whale | *Balaenoptera omurai* | 735 | 100% | |
| Striped dolphin | *Stenella coeruleoalba* | 735 | 100% | |

**Table S7.** Primers used in this study.

| **Primers’ name** | **Forward (5’-3’)** | **Reverse (5’-3’)** |
| --- | --- | --- |
| Pgli3^+^-dolphinZRS  Pgli3^+^-mouseZRS  Pgli3^+^-mouseZRSmut  Mouse-*HOXD13*  *FGF8*-Exon1-2  *FGF8*-Exon3  *FGF8*-Exon4  *FGF8*-Exon5  *FGF8*-Exon6 | ATTTCTCTATCGATAGGTACCATATTTGTGTGTATCCCTTT  ATTTCTCTATCGATAGGTACCGTGCTCATGTATGCTCCTGC  GCACAAAATCTGAGGTGACTTCCTCTCTTAATTAG  ATGAGCCGCTCGGGGACTTGGGACAT  ACATTCCCTGGGACCTGC  CAGAGGTACGGTGGCTGT  TGTCCGACTTTCCTGGGTG  GACTTTCCCAGGGTTGCACA  GCCTCCCCTCTGCCTCTGAT | ACTAATTGAGATGCAGCTAGCGAAACTGCTCAGTTTACATT  ACTAATTGAGATGCAGCTAGCAGATGTGCAAAGTTCAAGAA  CTAATTAAGAGAGGAAGTCACCTCAGATTTTGTGC  TCAGGAGACAGTGTCTTTGAGCTT  CTCTGTTCCTTAGTTCCCTCCT  TGGTGCGGCTGTAGAGTTG  GGAGGTGGAAAGGGAGATT  TGCCTTCTTCCTCCTTTGCT  GCTCCTCCCCAGCCTTCA |
