# Supplementary material for: Evolutionary genetics of flipper forelimb and hindlimb loss from limb development-related genes in cetaceans
Source: BMC Genomics. 2022 Dec 2;23:797. doi: 10.1186/s12864-022-09024-3 (PMC9719152; doi:10.1186/s12864-022-09024-3)
Supplement: Supplementary file 2 — Additional file 2: Fig. S1. Accelerated CNEs identified in our study. [file 12864_2022_9024_MOESM2_ESM.docx]

**Evolutionary genetics of** **flipper forelimb and hindlimb loss from limb** **development-related genes in cetaceans**

Linxia Sun**,** Xinghua Rong, Xing Liu, Zhenpeng Yu, Qian Zhang, Wenhua Ren, Guang Yang*, Shixia Xu*

Jiangsu Key Laboratory for Biodiversity and Biotechnology, College of Life Sciences, Nanjing Normal University, Nanjing 210023, China

* Corresponding to:

Shixia Xu, E-mail: xushixia@njnu.edu.cn (SX).

Guang Yang, E-mail: gyang@njnu.edu.cn (GY).

**Additional file 2**

**Figure Legends**

**Figure S1. Accelerated CNEs identified in our study.** Shown here are trees for seven examples of accelerated CNEs. Accelerated branches are marked with red; conserved branch and neutral branch are in green and purple, respectively. Redder branch indicates acceleration occurred at a higher rate or earlier on the branch, whereas greener one means later on the branch or no acceleration. Bayes factors (BFs) 1 & 2, the conserved (r1) and accelerated rate (r2) are listed below trees.
